# Relationship between suicide attempts of repetition and dependence to the cocaine: Report of clinical case

**DOI:** 10.1192/j.eurpsy.2021.1715

**Published:** 2021-08-13

**Authors:** M. Valtueña García, L. Lago García, R. Combina Fescina, A. Vázquez González, C. Ludwig, A. González Suarez, J. López Fernández, C. Huergo Lora, S. Ocio León, R. Arias Martino

**Affiliations:** 1 Psiquiatría, Hospital Vital Álvarez Buylla, Mieres, Asturias, Spain; 2 Psiquiatría, SESPA, Asturias, Spain; 3 Psiquiatría, Área VIII, Asturias, Spain; 4 Primary Care, Área VIII, Asturias, Spain; 5 Psiquiatría, Hospital Vital Álvarez Buylla, Asturias, Spain; 6 Psiquiatría, Drug Addiction Treatment Unit Area VII, Asturias, Spain

**Keywords:** Borderline personality disorder, dependence to the cocaine, suicide attempts of repetition, individualized terapeutic program

## Abstract

**Introduction:**

People with borderline personality disorder are at higher risk of repeating suicidal behavior. At the same time, numerous publications have demonstrated the relationship between cocaine dependence and suicide attempts of repetition.

**Objectives:**

Review the relationship between cocaine addiction, borderline personality disorder and repeated suicide attempts. Present through a clinical case the effectiveness of a comprehensive and multidisciplinary therapeutic plan with different mental health devices.

**Methods:**

To review the psychopathological evolution of a patient with a diagnosis of borderline personality disorder; dependence to the cocaine; Harmful alcohol consumption and suicidal behavior from the beginning of follow-up in mental health services to the present. Review the existing scientific evidence on the relationship between cocaine addiction and repeated suicide attempts. Analyze the eficacy of the different treatments available.

**Results:**

This is a longitudinal and retrospective study of the psychiatric history and evolution of a clinical case since the implementation of an individualized therapeutic program and the favorable results obtained. Intensive outpatient follow-up was carried out for high suicide risk and hospitalization in a psychiatric hospitalization unit, day care centre and therapeutic community.

**Conclusions:**

At present, the patient remains in abstinence with remission of suicidal ideation. The literature has shown the usefulness of intensive mental health follow-up programs to achieve remission of suicidal ideation and maintain abstinence from illegal substances.

**Disclosure:**

No significant relationships.

